# The second information revolution: digitalization brings opportunities and concerns for public health

**DOI:** 10.1093/eurpub/ckz160

**Published:** 2019-11-18

**Authors:** Martin McKee, May C I van Schalkwyk, David Stuckler

**Affiliations:** 1 Department of Health Services Research and Policy, London School of Hygiene and Tropical Medicine, London WC1H 9SH, UK; 2 Department of Policy Analysis and Public Management and Dondena Research Centre, University of Bocconi, Milan, Italy

## Abstract

The spread of the written word, facilitated by the introduction of the printing press, was an information revolution with profound implications for European society. Now, a second information revolution is underway, a digital transformation that is shaping the way Europeans live and interact with each other and the world around them. We are confronted with an unprecedented expansion in ways to share and access information and experiences, to express ourselves and communicate. Yet while these changes have undoubtedly provided many benefits for health, from information sharing to improved surveillance and diagnostics, they also open up many potential threats. These come in many forms. Here we review some the pressing issues of concern; discrimination; breaches of privacy; iatrogenesis; disinformation and misinformation or ‘fake news’ and cyber-attacks. These have the potential to impact negatively on the health and wellbeing of individuals as well as entire communities and nations. We call for a concerted European response to maximize the benefits of the digital revolution while minimizing the harms, arguably one of the greatest challenges facing the public health community today.

## An information revolution

Not for the first time, Europe is in the midst of an information revolution. Now it is digitalization. Before it was printing. The introduction of a printing press with moveable type by Johannes Gutenberg, around 1450, would have profound consequences for society. In a classic text, Briggs and Burke describe how the spread of print media challenged existing structures of power and hierarchy and encouraged a process of enquiry that would give rise to diversity of views.^1^ These developments fuelled the Enlightenment and the growth of knowledge that characterized it. They empowered people, allowing them to communicate radical ideas more effectively and to question accepted wisdom. But on occasions, the printed word was used for less benevolent purposes. In one of the earliest examples of what we might now call fake news, English pamphleteers printed scurrilous allegations about Marie Antoinette, primarily for the purposes of blackmail, in which they succeeded by extracting large sums from Louis XVI, while also contributing to the bloodletting that accompanied the French Revolution.^2^

Nowadays, print media continues to play these roles, but it is being increasingly replaced by digital media, in what has been described as the second information revolution.^3^ In some respects, this is just another way of disseminating words and pictures. In others, it is radically different. First, the advent of Web 2.0 allows anyone to publish content. This is no longer the preserve of those who own the printing presses. Second, and as a result, there is a massive expansion of the nature and quantity of information that is published. Once, people would confide their innermost secrets to their diary, to be shared, if at all, with a few close friends or after their death. Now, many aspects of their lives are recorded, in real time and in intimate detail, to the world. This includes data on their movements, tracked by geolocation services embedded in their mobile phones, their physiological parameters, collected by a growing range of wearable technologies, their interests and ideas, captured by tracking technology embedded in search engines and much else. Third, while a more open approach to publishing has brought huge benefits, exemplified by the knowledge contained within Wikipedia, it has also created many opportunities for those promoting disinformation. Finally, those who might once have read a printed book in private, for example, if it contained seditious material, risk losing that privacy, often within a matter of seconds. The content that is shared may also have implications for that individual when moving between countries with different approaches to legislating or censoring of social media platforms.^4^ Some risk being targeted for their presumed political views, whether by a repressive state or by an algorithm that uses their interests to direct information to them, reinforcing their existing beliefs and polarizing attitudes within society.

## Benefits of digitalization

These developments have important implications for health. Many are beneficial. Thus, the growth in digital information can contribute to generating and sharing of knowledge. Those with rare diseases can come together over vast distances, creating a community that can share experiences and insights. Patients with chronic conditions can become much better informed about their disease, including ways of adapting to its impact on them.

Patients (and the public) can also contribute their data to improved understanding of disease, including insights into aetiology, diagnosis and possible avenues for treatment. For example, monitoring of Internet traffic is being used to provide early warning of disease outbreaks.^5^ When linked to other data, such as patterns of mobility obtained from mobile phone records or meteorological data, it can even enhance models used to predict outbreaks.^6^^,^^7^ Clinical data, drawn from large populations, can be used by artificial intelligence applications to discern patterns, thereby improving prognostic tools.^8^ Clinical data can also be used to feed into machine learning applications that allow automation of some diagnostic processes, especially those involving image processing, in areas such as pathology, radiology, dermatology and ophthalmology.^9^ New forms of wearable technology and other mobile devices may offer opportunities for disease prevention, although so far rigorous evidence of their effectiveness is lacking.^10^

Analysis of internet searches, which offer pointers to issues that are concerning people in real time, is increasingly being used to provide clues to emerging health trends long before they become apparent in traditional data sources. Thus, internet searches for suicide-related terms, which can be obtained almost instantaneously, correlate with actual suicides in young people, for which data may only become available after several years.^11^ The uses of Internet search data are limited only by the imagination of researchers.^12^ For example, a concern about a possible drug interaction was supported by the finding that people in widely scattered locations had been searching in combination for the products involved.^13^ Searches can also reveal things that individuals might be reluctant to disclose in research using more traditional methods. An example is the identification of an association between racism in the USA, captured by searches for the ‘N-word’ and black/white disparities in mortality.^14^ Another example is a study that showed how discussion of Adderall, a stimulant used widely by university students in the USA, peaked during exam periods and was concentrated in communities hosting leading universities.^15^

Monitoring of social media can offer insights into how people understand health related conditions, using content and sentiment analysis to highlight how people understand key issues, thereby informing the development of health promotion material, as well as identifying and responding to key influencers,^16^ including those celebrities who are paid to promote health damaging products.^17^

On a more practical level, advances in digital technology provide new ways of interacting with health services, including direct bookings of appointments, ordering repeat prescriptions and diagnostic kits e.g. for HIV self-testing and in some cases remote consultations or real-time surgical advice using packages such as Skype. There is also growing recognition of the potential benefits and risks of using digital messaging apps, such as WhatsApp, to facilitate communication within the healthcare setting as well as for enhancing medical education.^18^

## Five concerns about digitalization

There are, however, some concerns and in some cases, threats posed by the digital revolution. One is whether health systems have the capacity, in terms of human resources and governance structures, to take advantage of the opportunities set out above, as discussed in a recent report by the UK Health Foundation.^19^ But beyond that, we can identify five issues that require attention: discrimination; breaches of privacy; iatrogenesis; disinformation and misinformation or ‘fake news’ and cyber-attacks. The list is far from exhaustive but serves to illustrate the scale of the challenges facing the public health community in this digital revolution.

Among the more benign is the potential for inadvertent discrimination. This can occur when an algorithm replicates human behavior that, consciously or unconsciously, discriminates on grounds of, for example, ethnicity. In one highly cited study, a computer was programmed to learn English by trawling through vast quantities of text. It learnt to associate male names with career-related terms and female names with family-related terms, European names were associated with pleasant terms and African–American names with unpleasant ones.^20^ Problems can also arise from use of unrepresentative data to generate the algorithms. A standard database used to develop commercial facial recognition tools in the USA underrepresents females and people with dark skin with the resulting tools perform much poorer with faces of dark skinned females.^21^ This can have important consequences when, for example, innocent individuals are misidentified as wanted criminals.

There is also scope for intentional discrimination.^22^ The US investigative journalism organization ProPublica demonstrated that they could restrict advertisements on Facebook for attractive rental properties in New York to exclude African–Americans, Jews and anyone who had shown an interest in aids for disabled people.^23^ ProPublica has also revealed that TurboTax manipulated Google and other search engines through the use of coding, essentially deceiving lower-income Americans into providing a payment for filing of their taxes despite being eligible to do so free of charge.^24^ In these ways, digitalization can widen inequalities.

Finally, there is a concern that interventions based on eHealth may be taken up most by those whose needs are least,^25^ while those who are unfamiliar with new technology, such as some older people, become resentful at being excluded from these advances.^26^ As with many information interventions, there is a risk that eHealth could widen inequalities in health between those with high and low education.^27^

The second area of concern is the potential for breaches of privacy. Public health researchers face formidable obstacles when conducting surveys, especially when studying sensitive issues. Yet at the same time, social media companies are gathering vast quantities of information about their users, including not only what is posted on their particular platform, but information harvested from numerous linked sources, including geolocation. Advances in machine learning now make it possible to assemble extremely detailed profiles of many aspects of the lives of those who have any significant online presence.^28^ The scale of this activity was revealed by investigations into the use of precisely targeted advertisements on Facebook during a number of electoral events, including the 2016 US Presidential election, the UK’s EU referendum and elections in Kenya and South Africa. This also relates to the previous point, as only those who are the intended recipients of the messages may see them, so that those seeking to counter disinformation, considered further below, may be unaware of them. These tactics have often been used to advance causes or parties that oppose public health policies.

In recent years, companies have exploited the benefits (to them) of digital transactions, assisted by the increased use of credit and loyalty cards. One notorious example, described in a detailed, although now dated review of marketing methods, was where a company sent a teenager pregnancy related material having predicted her condition from her purchases, before she had disclosed her condition to her parents.^29^ This is an area where progress is extremely rapid, assisted by techniques such as facial and voice recognition. For example, Amazon has patented the concept of analyzing user’s moods, facilitated by the continuous monitoring of conversations by its Alexa device, so as to allow targeted advertisements adjusted to how one is feeling.^30^ The increased use of methods such as these have clear implications for those researching the emerging field of corporate determinants of health.^31^ Indeed, the social media companies are now becoming the subjects of public health research themselves, studied by those seeking to understand the influence they and their clients, wield. Companies such as Facebook, Google and Twitter, hold an effective monopoly on their technology. While governments increasingly recognize the need to address this issue, what constitutes an optimal approach continues to be debated.^32^

The third concern is what has been termed ‘e-iatrogenesis’, defined as ‘patient harm caused at least in part by the application of health information technology’.^33^ This is not new. The advent of more sophisticated imaging techniques has led to many people undergoing complex invasive investigations for ‘anomalies’ that we now know are simply variants of normal anatomy. As noted above, machine learning and artificial intelligence hold considerable potential in the field of diagnosis, especially where it is important to be able to recognize complex patterns.^34^^,^^35^ However, it is essential that claims made by those promoting these approaches are evaluated, just as any other form of health technology. In particular, there is a risk of unintended consequences. For example, when skilled human observers were presented with images already annotated by computers their accuracy was reduced.^36^ Particular criticism has been directed at an app being promoted within the English National Health Service, with examples circulating on social media of some frankly bizarre and, in some cases potentially dangerous advice, leading to complaints to the medical device regulator.^37^ There is also considerable potential for companies making health-related items to design apps that promote their products, in a way that is analogous to the way that pharmaceutical companies have sought to stretch the definition of disease, in some cases manufacturing new ‘illnesses’.

The fourth concern is that what is spread on digital media can be seriously misleading. A recent UK Parliament report on ‘fake news’ identified two different types of incorrect information,^38^ disinformation and misinformation. Disinformation is defined as that which is intentionally designed to mislead. Vaccination has been among the most intensively targeted issues, to the extent that it was selected by the US Department of Defence for a competition to find the best way to identify ‘influence bots’.^39^ Much of the online content on vaccination is misleading and incorrect messages are consistently found to be liked and shared more often than those that are accurate.^40^^,^^41^ But where do these messages come from? One very detailed study of vaccine-related posts on Twitter provides some answers.^42^ It found three types of account that were especially likely to spread vaccine-related disinformation. One was Russian trolls, identified from lists compiled by US authorities that point to links with the Russian Internet Research Agency, an organization implicated in electoral interference in several countries, including the USA and UK. Using the hashtag #VaccinateUS, they disseminated pro- and anti-vaccination messages. This was consistent with their approach to other issues, such as gun control and the #BlackLivesMatter movement, with messages that differed from other sources by linking vaccines with particularly divisive topics such as race and religion. The second comprises - to be consistent with below what were termed ‘sophisticated bots’, which automatically promote particular types of content. Again they included pro- and anti-vaccine messages, often with the apparent goal of encouraging people to believe that the medical community is divided. A third group comprises ‘content polluters’, using anti-vaccine messages that encourage curiosity. Many of these are used to malware or act as clickbait, directing readers to sites that generate income. A subsequent paper by some of the same authors provides a comprehensive taxonomy of the diverse range of malicious actors on Twitter.^43^

Clearly it is necessary to respond to the viral spread of mis- and dis-information, but this is not always easy. For example, challenging incorrect anti-vaccine beliefs held by those who believe that the pharmaceutical industry manipulates data is not made easy by a few high profile cases where they have been shown to do so. Some responses could perversely ‘backfire’,^44^ paradoxically, increasing the propensity for some people to believe the false message.^45^^,^^46^ It may be more effective not to engage with the details but instead appeal to values. Similarly, it is important not to suggest that a belief that is actually uncommon is widely held.^47^ These are just a few examples of what is now a rapidly growing body of research that public health professionals will need to become familiar with.^48^^,^^49^

The public health community must also brace itself for online attacks, with concerted campaigns of often deeply unpleasant and personal abuse.^50^ Public health organizations and training bodies should therefore seek to train their staff to be effective advocates using social media while providing them with the support and guidance on how to do so safely.

The final concern considered here, and one that could become a serious threat to health at an individual level, relates to the risk that machine learning applications will be subject to adversarial attacks. This can take several forms. As these applications continuously learn from the data inputted to them, there is scope to influence the algorithms with fraudulently manipulated inputs, which can be in the form of written or audible text or images.^51^ These could be undertaken for many different purposes, such as attempted extortion from the owner of a diagnostic system, although most attention has been directed at the potential to circumvent systems for authorizing treatment for billing in the US health system. Thus, one recent paper showed how easy it was to manipulate the pixels in an image of a mole to change the automated assessment of whether it was likely to be benign or malignant, with the change imperceptible to even skilled observers. Another form of attack was the use of the Wannacry ransomware, which temporarily paralyzed large parts of the English National Health Service in 2017.^52^

## Conclusions

As this brief review shows, the digital revolution provides opportunities to improve health, but also threats. We have witnessed profound expansion of opportunities to share and access information and experiences, to express ourselves and communicate with each other 24 h a day and over vast distances. This has simultaneously led to a marked increase in opportunities to manipulate and deceive, thereby rendering people’s health and wellbeing vulnerable to novel threats. Maximizing the benefits while minimizing the harms is arguably one of the greatest challenges facing the public health community. Crucially, there is a need to address the power imbalances and inequalities that determine who benefits and who is harmed. Open and transparent discussion is essential to begin addressing these issues. We hope that this paper will contribute to this dialogue and encourage robust responses, including governance structures that are appropriate for these emerging challenges, just as there are for other health issues. No one country can tackle these issues on its own. The European Commission’s Expert panel on Investing in Health has recently published a detailed report on digitalization and health, which provides recommendations on how to maximize the opportunities and minimize the harms.^53^ This is a good start but continued concerted action by the European institutions, drawing on the expertise and authority in health and information technology sectors, working with national and European agencies engaged in intelligence and cyber security, will be essential.

*Conflicts of interest*: None declared.
